# Biomechanical findings in horses showing asymmetrical vertical excursions of the withers at walk

**DOI:** 10.1371/journal.pone.0204548

**Published:** 2018-09-27

**Authors:** Anna Byström, Agneta Egenvall, Lars Roepstorff, Marie Rhodin, Filipe S. Bragança, Elin Hernlund, René van Weeren, Michael A. Weishaupt, Hilary M. Clayton

**Affiliations:** 1 Department of Anatomy, Physiology and Biochemistry, Faculty of Veterinary Medicine and Animal Science, Swedish University of Agricultural Sciences, Uppsala, Sweden; 2 Department of Clinical Sciences, Faculty of Veterinary Medicine and Animal Science, Swedish University of Agricultural Sciences, Uppsala, Sweden; 3 Department of Equine Sciences, Faculty of Veterinary Medicine, Utrecht University, Utrecht, The Netherlands; 4 Equine Department, University of Zurich, Zurich, Switzerland; 5 Department of Large Animal Clinical Sciences, East Lansing, Michigan, United States of America; University of Illinois, UNITED STATES

## Abstract

The walk and trot are inherently symmetrical gaits, making them potentially suitable for the detection of left-right asymmetries. The aims of this study were to describe asymmetrical vertical excursions of the withers at walk in non-lame high-level dressage horses and to seek associations between these asymmetric movements and other kinematic variables and vertical ground reaction forces (vGRFs). Seven dressage horses, judged clinically as being sound, walked unridden and unrestrained on a treadmill with an integrated force measuring system (480 Hz), from which spatiotemporal and vGRF variables were extracted. Markers were tracked by 12 infrared cameras (240 Hz). The vertical position of the sixth thoracic vertebra (T6), limb protraction and retraction distances throughout stance, and global limb lengths were determined. Contralateral trial-mean differences were calculated, including difference in T6 minimum vertical position between contralateral steps (T6minDiff). Mixed models were used to study associations between symmetry parameters. Trial-mean T6minDiff ranged between 0.3–23 mm. Of the seven horses, five consistently dropped the withers more in early left forelimb stance, one was fairly symmetrical, and one dropped the withers more in early right forelimb stance. Comparisons between contralateral limbs showed the following associations. The forelimb that was retracted when T6min was lowest showed greater retraction at toe-off (1 mm increase predicted 0.17 mm T6minDiff increase) and shorter stance duration (1 ms decrease predicted 0.3 mm T6minDiff increase). The hind limb that was in midstance when T6min was lowest showed a greater range of motion during the stance phase (1 mm increase in protraction or retraction predicted 0.2 mm T6minDiff increase). The haunches were displaced away from the side of the forelimb that was protracted when T6min was lowest (1 mm lateral shift predicted 0.07 mm T6minDiff increase). Forelimb and hind limb vGRF parameters were non-significant. Asymmetry of vertical withers movement in horses assessed as being sound at trot was related to a complex pattern of asymmetries in spatiotemporal variables throughout the stride cycle rather than to vertical load redistribution between the forelimbs. This suggests that the asymmetry may be due to inherent laterality rather than weight-bearing lameness.

## Introduction

The walk and trot are inherently symmetrical gaits, which makes them potentially suitable for the detection of left-right asymmetries in spatiotemporal and ground reaction force (GRF) variables. Detection of such asymmetries is relevant both in veterinary medicine and in equestrian sports, especially dressage. Lameness is typically associated with kinematic asymmetries that result in redistribution of vertical ground reaction forces (vGRF) from the lame limb(s) to the compensating limbs. The trot is the preferred gait for lameness detection [1,2], because the presence of a suspension phase results in higher vGRF compared to the walk [3]. Peak vGRF and vertical impulse are the most reliable indicators of weight bearing lameness [4–6].

Although the gait is regarded as being less important for lameness diagnosis, the walk is of great importance in a number of equestrian disciplines. This is exemplified by the fact that all current international dressage tests award a double coefficient for the quality of both collected walk and extended walk [7]. Symmetry and regularity are important criteria for judging overall gait quality and hence for dressage performance. Horses exhibiting irregularity in the walk may even be eliminated from competitions and, consequently, be presented for clinical work up. This warrants the study of asymmetries in the walk of non-lame dressage horses.

With the increasing availability of equipment capable of accurate quantification of equine gait [8], the measurement of even subtle asymmetries has become possible. However, this poses new problems for the interpretation of the data since asymmetry and lameness are not interchangeable terms [9]. No living being is perfectly symmetrical; locomotor asymmetries may be the result of non-pathological conditions such as cerebral laterality, which is manifested as a motor dominance of the left/right side of the body [10]. This type of sidedness is known to be present in many animal species [11] and may be associated with low level kinematic and GRF asymmetries. Motor laterality in horses has, for example, been studied in terms of preferred canter leads [12], kinematic asymmetries in the gait of young Standardbred trotters [13] and a preference for grazing or halting with one forelimb in a more advanced position [14]. Sound horses exhibit left-right asymmetries in axial (twisting) moments around the fore hooves when walking on straight lines [15,16] and on circles [17] and these have been interpreted as manifestations of sidedness.

Several studies have focused on non-pathological sources of asymmetry at trot [1,18–19], in which the vertical excursions of head, withers and/or pelvis are commonly used for symmetry evaluation. Withers symmetry has been shown to be the most direct indication of asymmetry in the forelimbs that is least prone to confounding influences [20]. However, the walk has been largely neglected and relatively little is known about inherent asymmetries in this gait. Our objective was to determine the amount of asymmetry of the vertical excursions of the withers, in a group of non-lame high-level dressage horses walking freely on a treadmill. This was performed using kinematic and kinetic data from an earlier study [21]. For any systematic and consistent asymmetry pattern that was identifiable at group level, mixed-models were used to determine which asymmetries in vGRFs, and/or inter-limb timing, and/or limb kinematics that best predicted the asymmetric withers movement at the walk. We used an approach that involved plotting graphs and performing mixed-effects modelling on data from stride- and trial-level and aimed at obtaining stable results from the models that were congruent with conclusions drawn from graphical representations of raw and descriptive data.

## Materials and methods

The Animal Health and Welfare Commission of the canton of Zürich (188/2005) approved the experimental protocol.

### Horses

Seven Warmblood horses (mean±SD height: 1.70±0.07 m) that were competing in high-level dressage competitions were studied. Before the start of the study, all horses were deemed clinically sound at trot by an experienced veterinarian (MW) and were accustomed to treadmill exercise using a standard protocol for treadmill habituation.

#### Data collection and management

The horses walked and trotted unridden in a free unrestrained head and neck position on a treadmill (Mustang 2200, Graber AG, Fahrwangen, Switzerland) equipped with an integrated force measuring system [22]. A handler was positioned in front of the horse. Trials of 12s duration were recorded at walk and trot at a series of increasing speeds within each gait. Trot data were used only for comparison of vGRF symmetry with walk data.

A full-body set of spherical, 19 mm diameter markers were glued to the skin over anatomical landmarks on the head, neck, trunk, and limbs (S1 Fig). Marker positions were tracked by 12 infrared optical motion capture cameras (ProReflex, Qualisys, Gothenburg, Sweden) sampling at 240 Hz. Q-Track software (QTrack, Qualisys, Gothenburg, Sweden) was used to capture data. The instrumented treadmill sampled vGRF at 480 Hz. The treadmill software automatically detected the hoof positions during stance and decomposed the reaction force responses at the multiple bearing points of the treadmill platform into vertical forces acting on each of the four hooves. For each stride and limb, first contact and toe-off were determined by the intersection of the linear approximation of the initial and terminal slope of the force curve with the zero-baseline [22].

Raw 3D coordinates describing the marker locations, and spatiotemporal and vGRF data, were exported to and managed in Matlab (Matlab version 2016b, The Math Works Inc., Natick, USA).

### Kinetic and spatiotemporal variables

The following spatiotemporal and vGRF (normalised to horse body mass) variables were extracted from the treadmill force-measuring system and were used to develop the statistical models:

1. stance duration and stance length (distance traveled by the hoof on the treadmill belt during stance) of the individual limbs,
2. longitudinal position of each hoof on the treadmill at first contact and toe off. The positive direction was towards the rear of the treadmill,
3. duration of bipedal (diagonal, ipsilateral) and tripedal support phases, measured as a percentage of stride duration (% SD),
4. time of first and second vGRF peaks in the forelimbs and hind limbs measured as a percentage of stance duration (% StD),
5. peak vGRF magnitude (normalised to horse body mass) for the first and second force peaks,
6. transverse distance between placements of the ipsilateral fore and hind hooves, i.e, ipsilateral limb tracking. Positive values indicate that the hind hoof is placed to the right of the ipsilateral fore hoof. Negative values indicate that the hind hoof is placed to the left of the ipsilateral fore hoof.

### Kinematic variables

The analysis used markers on the midline overlying T6, T10, T13, and S3, and limb markers on the tuber spinae scapulae, the elbow joint and the lateral walls of all four hooves. The variables were T6 vertical position; fore and hind limb protraction and retraction distances measured as the distance along the longitudinal-horizontal axis from T6 to the fore hoof marker or from S3 to the hind hoof marker; and limb lengths measured as the global (Euclidian) distance from T6 to the fore hoof marker or from S3 to the hind hoof marker.

Data for the stance phase of each limb was time-normalized to 101 data-points, and the time of T6 minimum vertical position (T6min), which occurs early in the stance phase of each forelimb, was determined. Global limb lengths and longitudinal T6/S3-hoof distances for the left and right limbs were determined at the time of each T6min, i.e. two values for each limb, when the forelimb was in early stance and late stance, and when the hind limb was in midstance and midswing.

### Calculation of left-right differences

Contralateral differences were calculated for all kinetic and kinematic variables (left side values minus right side values). For the ipsilateral limb tracking difference (model vi), a positive value indicated that the haunches were tracking to the right of the shoulders. Trot vGRF peak forces were extracted from the treadmill data and differences between contralateral limbs were expressed as a percentage of left-right mean (100*[left—right]/[left + right]*0.5.

For the kinematic variables differences were calculated as values at T6min in early LF stance minus contralateral values at T6min in early RF stance. For example, the global distance from T6 to the LF hoof at the time of T6min with the LF in early stance was compared to the value from T6 to the RF hoof at T6min in the early RF stance.

For the longitudinal hoof positions on the treadmill at the start and end of stance and in the kinematic model, a positive difference in limb protraction-retraction at T6min means that the left limb is caudal to the right hind limb, i.e. the left limb is relatively less protracted or more retracted, at this instant. Because the value at RF T6min was subtracted from the value at LF T6min, a positive difference value for the retracted forelimb and the hind limb in swing means that the left limb is located more caudally at the time of RF T6min compared with the right limb at LF T6min.

### Statistical procedures

Given the large number of kinematic markers (S1 Fig) and kinetic variables available [21], a systematic variable selection process was undertaken, with plotting of raw data series and screening for visual associations between variables. This was followed by mixed models analysis of left-right differences based on data calculated at the level of the individual strides/steps (stride-level data) or averaged across all strides in a trial (trial-level data). Because the movements of anatomically-linked segments are naturally associated, many statistically significant relations can easily be found which may or may not be relevant to the questions of the study. Care was therefore taken to model variables that graphically suggested a relatively strong association to T6min, or where biomechanical reasoning suggested a possible association. When in doubt, horse-specific models on stride-level data were made to confirm that our results were consistent both on individual level and group level (data not shown).

Final models were made on trial-level data to avoid spurious statistical associations. The models were developed on sets of variables with possible associations to each other (*i*.*e*. models i-vi for the kinetic variables and one model for the kinematic variables). This approach did not overtly introduce collinearity in the modelling process.

Descriptive statistics, including Pearson correlations to demonstrate associations, were made. Mixed models were used to study associations in SAS (PROC MIXED, SAS version 9.4, SAS Institute Inc., Cary, North Carolina). For both kinetic and kinematic models, we used the difference between T6 minima at LF and RF early stance as outcome variables (T6minDiff). The outcome variable was square-root transformed to achieve reasonably normally distributed residuals. Models were made on trial-mean data. The horse ID was used as a random effect. The covariance structure was set to variance components. The linearity of independent fixed effects was checked in two ways. The first was through plotting. The second was through modelling: to each univariable fixed-effect model (one fixed effect at a time) the squared variable was added and checked for significance. The p-value limit was 0.05 throughout, as we diminished the power by using mean trial-level data (instead of stride-level data). Within-limb interactions were tested in kinematic models, but further interactions were ignored due to difficulties with interpretation. Residuals were examined for homoscedasticity and normality. The Akaike criterion was used to guide model selection when appropriate. After using the square-root transformed variable for selecting significant variables, the models were remade on the untransformed scale so that the findings were expressed in SI standard units that are more easily understood. This was considered possible, as data were relatively close to normal (the square-root transformation is adjacent to no/unity transformation on the ladder of powers [23]).

For the kinetic and spatiotemporal analyses using data from the treadmill force-measuring system, six models were made for each set of variables (i-vi). After this, the significant terms from these models were tried together in a combined kinetic model. Finally, one combined kinetic and kinematic model was made from the final kinetic and kinematic models.

## Results

### Descriptive

There were 37 walk trials, between 5 and 8 trials per horse, and with 8–14 strides per trial. Speed varied from 1.18 m/s to 1.78 m/s, with median speed over all trials being 1.56 m/s. There were 32 trot trials, between 4 and 6 trials for each horse. Each trial had data for 22–26 strides. The speed varied from 2.68 m/s to 3.69 m/s, median speed over all trials was 3.10 m/s.

**Fig 1:**
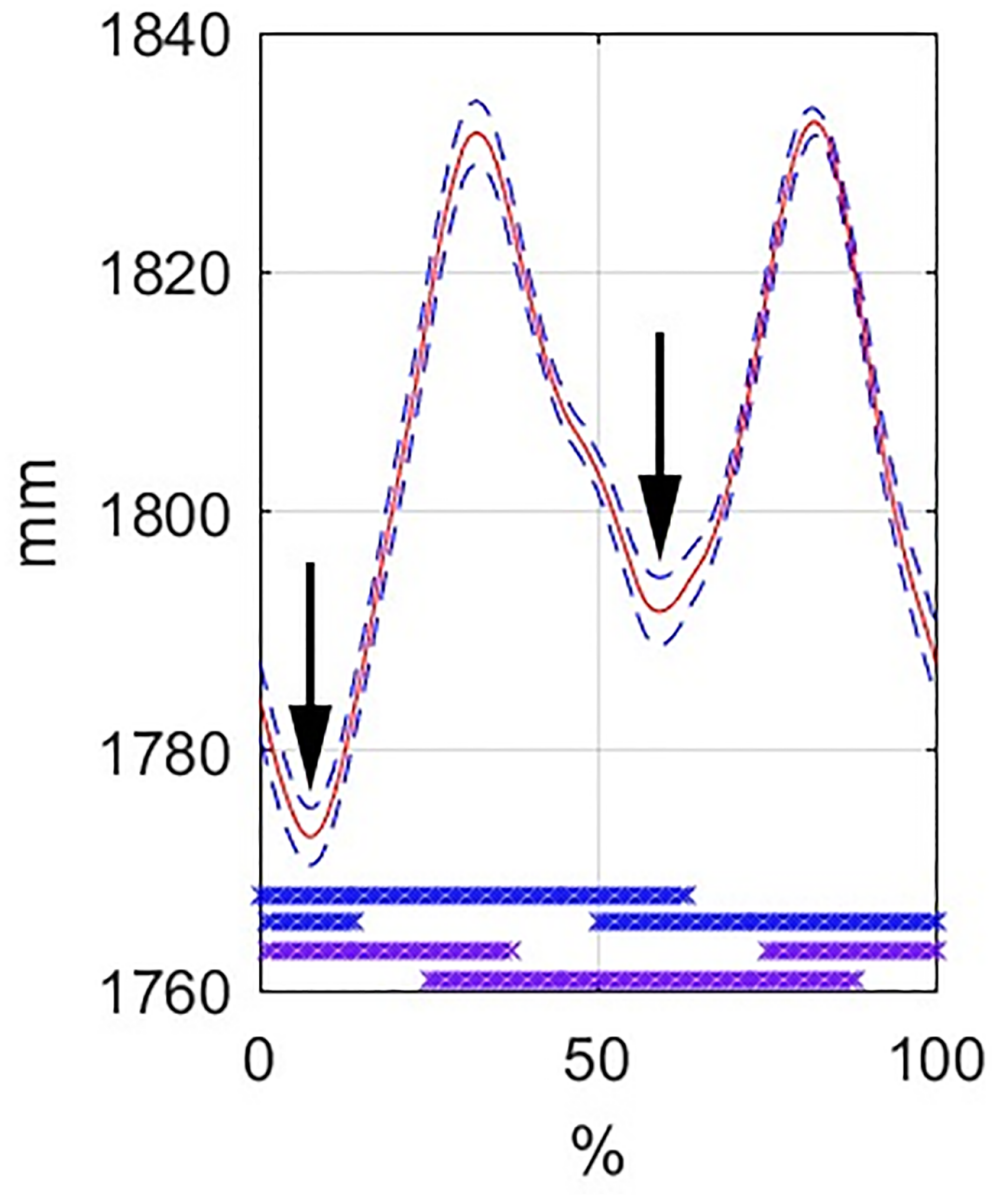
T6 vertical position in walk at 1.58 m/s in horse 2. Normalised (0–100% of stride) mean-stride curve (red line) +/- standard deviation (blue interrupted line) for vertical T6 position. Bars at the bottom of the graph indicate stance for the left fore and right fore (blue colour), left hind and right hind limbs (purple colour), from top to bottom. Arrows indicate minimum T6 vertical position in early left and right forelimb stance.

T6 vertical position shows two cycles per walk stride; the minima occur during forelimb overlap when one forelimb is protracted and the other is retracted (Fig 1). The maxima coincide with forelimb mid-stance. Raw data for T6 vertical position and fore and hind vGRF were plotted against time and, for the trial with median speed in each horse, the graphs are shown in S2 Fig. Based on visual evaluation of raw data from all available trials (in S2 Fig, not all data shown), two horses (1 and 5) showed a slight difference between the T6 minima during early stance of the LF and RF, and one horse (3) showed a moderate difference, while the remaining horses (2, 4, 6 and 7) showed a more substantial difference. In S1 Video the withers movement is shown for a typical example (horse 2, 1.58 m/s, shown at 50% reduced play speed). This horse drops the withers more in early stance of the left forelimb, compared to early stance of the right forelimb.

**Fig 2:**
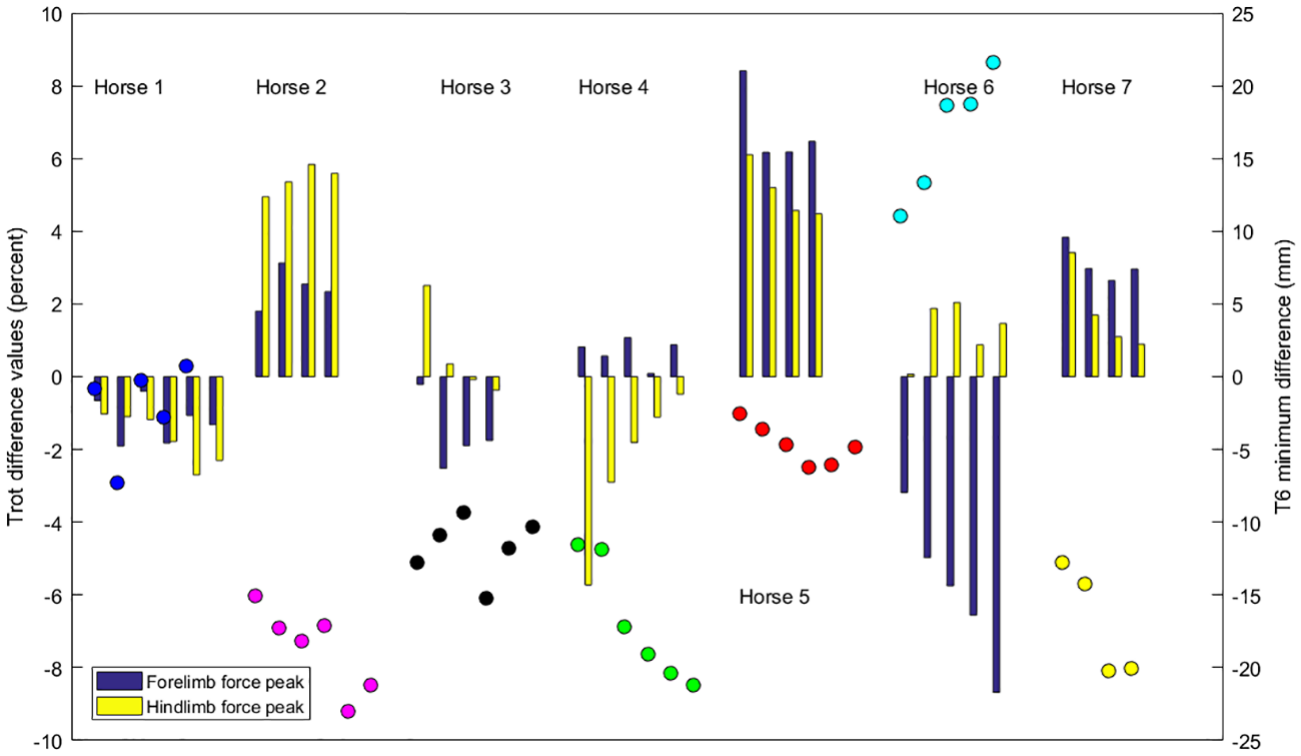
Comparison of contralateral differences in vertical ground reaction forces at trot and vertical T6 minimum positions differences at walk. Contralateral differences are calculated by subtracting the value for the right side from that of the left side. The bars represent differences per trot trial, expressed as percent left-right mean value for forelimb peak vertical force (blue) and hind limb peak vertical force (yellow). Each colored dot represents T6 minimum difference (mm) for the individual trials: Horse 1: blue; Horse 2: magenta; Horse 3: black; Horse 4: green; Horse 5: red; Horse 6: cyan; Horse 7: yellow.

Fig 2 presents data on trial-mean T6minDiff in the walk trials together with asymmetries in vGRF variables in trot trials, expressed as left-right differences. Only horse 1 appears symmetrical in both walk kinematics and trot vGRFs. Horses 2, 5 and 7 show the same pattern with higher vGRF values for the LF and left hind (LH) limbs in trot and they drop the withers more at the beginning of LF stance at the walk. However, horse 5, which has the highest asymmetry in trot, has only a small T6minDiff at the walk. Horse 6 shows the opposite pattern with higher vGRF values for the RF at the trot and drops the withers more at the beginning of RF stance at the walk. Horse 4 deviates from the pattern of the other horses by showing higher vGRF values for the right hind limb (RH) at trot, but dropping the withers more on the LF in walk. However, it is worth noting that the vGRF differences are not consistent in magnitude between trials in this horse.

The correlation between vertical positions of the thoracic midline markers was high (S3 Fig; horse-trial median Pearson correlations between T6-T10 ranged from 0.67 to 0.99, group median 0.92, and T6-T13 from 0.55 to 0.97, group median 0.86). This makes it unlikely that asymmetric marker placement or other marker-related errors were responsible for the asymmetrical T6 vertical pattern. S2 Fig also shows raw data for the vertical positions of T6, T10, T13 and left-right means of the vertical positions of the tuber spinae scapulae and elbow markers. Visual inspection of these plots suggests a consistent association between the left-right stance differences in vertical minima of these variables.

**Fig 3:**
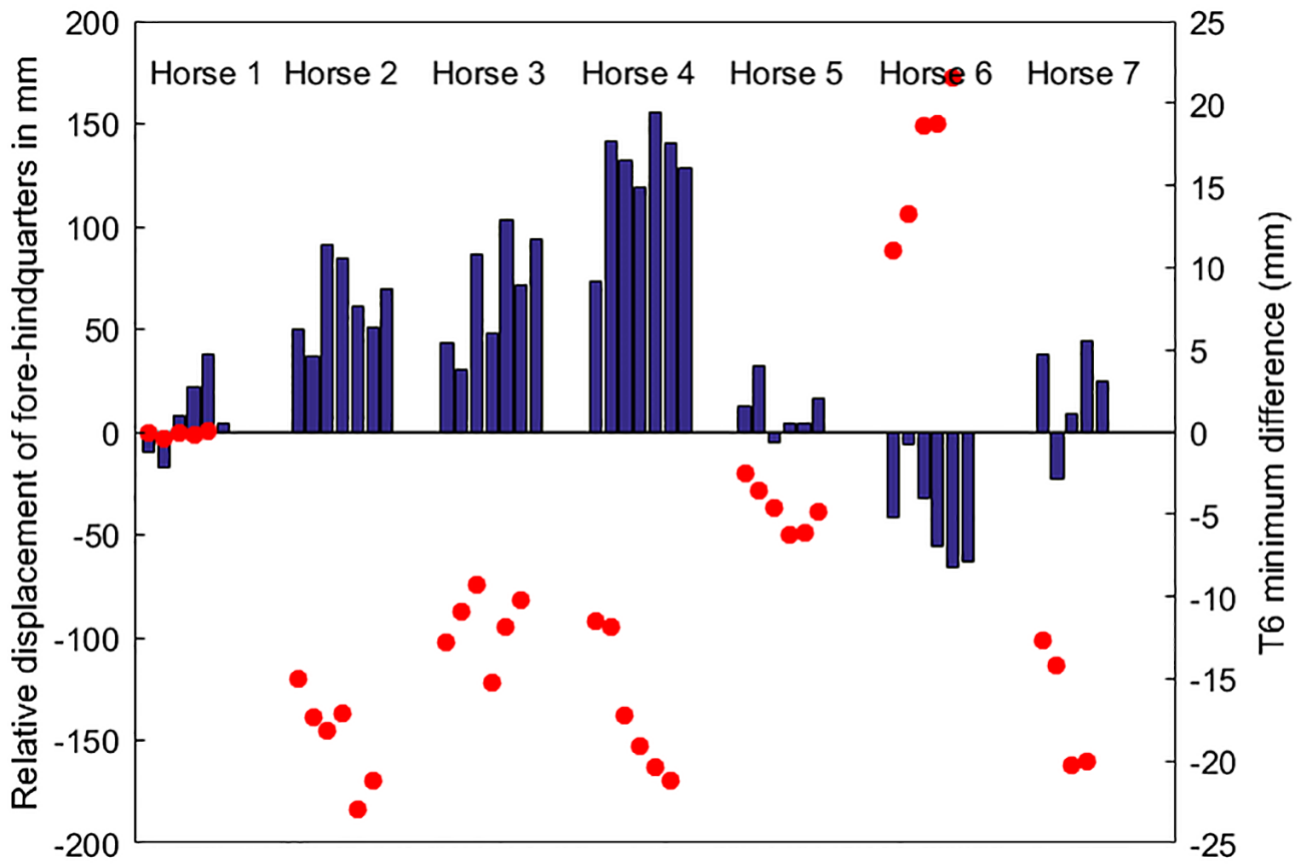
Hind limb tracking relative to the ipsilateral forelimb in a transverse direction and T6 minimum vertical position differences on a trial by trial basis at walk. The bars represent differences in position of the hind hoof versus the ipsilateral fore hoof placement in a transverse direction. Values are mean of the differences within each (left and right) ipsilateral limb pair for each walking trial. The red dots are T6 minimum differences for the corresponding trials. The trials within each horse are arranged from left to right in order of increasing speed. A positive ipsilateral limb tracking value indicates that the haunches are to the right of the shoulders.

The lateral deviation of the haunches relative to the shoulders is represented in Fig 3. The vertical bars indicate deviation of the hind quarters in relation to the forehand in the transverse direction (i.e. ipsilateral limb tracking difference). Horses 2, 3 and 4 are strongly positive in all trials and horses 1, 5 and 7 are weakly positive in the majority of trials indicating that the hind hoof print is displaced to the right of the ipsilateral fore hoof print. In most horses the withers are lower in early LF stance and the hind limbs deviate to the right. Only horse 6 shows the opposite pattern with negative values in all trials indicating that the hind hoof prints are to the left of the ipsilateral fore hoof prints and the red dots indicate that the withers are lower when the RF is in early stance in this horse.

### Models

**Fig 4:**
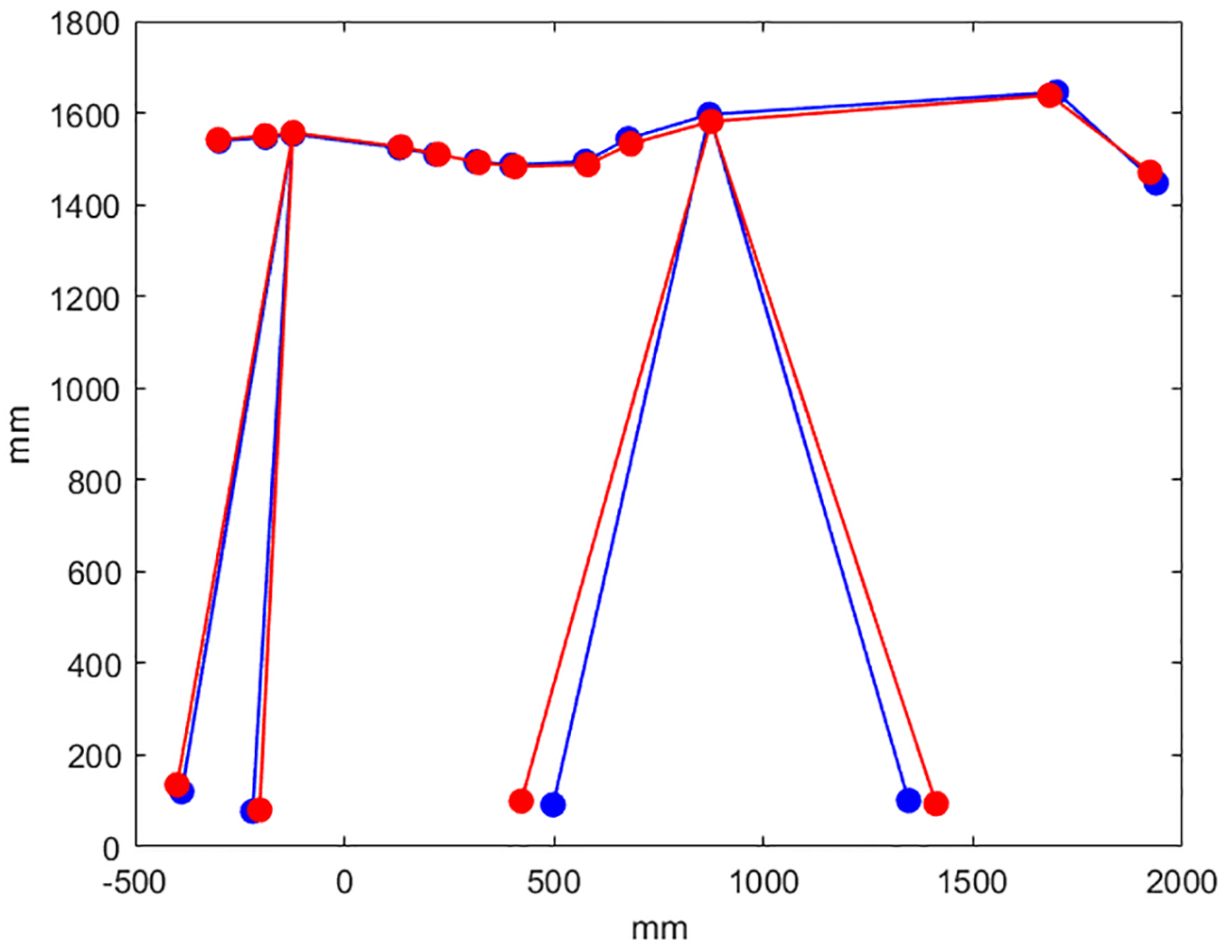
Schematic illustration of kinematic model results in one horse. The stick figures illustrate the limb positions at the moments of T6 vertical minima during the early stance phases of the left and right forelimbs for horse 3 with velocity 1.58 m/s. Red/blue lines indicate body segment positions at the lower/higher T6 vertical minimum.

The independent variables that were tested plotted against T6MinDiff are shown in S4 Fig, kinematic model findings are illustrated schematically in Fig 4, and S1 Table contains the dataset. Variable squares remained in a few preliminary models, but none of them improved Akaike’s criterion, and they were thus omitted. None of the within-limb interactions that were tried in the kinematic model was significant. Residual plots of transformed models were considered good or occasionally adequate. Table 1 shows the resulting models re-run on untransformed data (for improved interpretability of the magnitudes of the estimates) and the p-values from the transformed models. In most cases, the p-values from the transformed models are lower than those from the untransformed and presented models.

**Table 1:** Model estimates from the mixed models in the study.

		Kinetic models and kinematic model			P-value	
Line id	Symmetry variables	Est	SE	95% CI	Untrans.	Transf.
1	Intercept	-6.74	4.14	(-15.1, 1.4)	0.15	<0.0001
2	Forelimb stance length (mm)	0.28	0.09	(0.1, 0.5)	0.007	0.004
3	Hindlimb stance length (mm)	0.16	0.07	(0.1, 0.3)	0.03	0.004
4	Forelimb stance duration (ms)	-0.30	0.14	-(1.1, 0.0)	0.04	0.01
5	Intercept	-6.04	3.37	(-12.6, 0.6)	0.12	<0.0001
6	Hindlimb stance protraction (mm)	-0.20	0.07	(0.1, 0.3)	0.007	0.0009
7	Forelimb stance retraction (mm)	0.17	0.05	(0.1, -0.1)	0.001	0.002
8	Hindlimb stance retraction (mm)	0.20	0.07	(0.1, -0.1)	0.01	0.003
9	Intercept	-7.51	4.35	(-16.0, 1.0)	0.14	<0.0001
10	Ipsilateral support duration (% of SD)	3.82	1.13	(2.1, 11.3)	0.002	0.004
11	Intercept	-4.95	3.86	(-12.5, 2.6)	0.25	<0.0001
12	Ipsilateral limb tracking (mm)	-0.07	0.02	(0.1, 0.0)	0.006	0.01
13	Intercept	-1.63	3.26	-(8.1, 4.8)	0.64	<0.0001
14	T6-hoof global distance protracted fore (mm)	0.29	0.11	(0.1, 0.5)	0.02	0.005
15	T6-hoof global distance retracted fore (mm)	-0.45	0.12	(-0.7, -0.2)	0.001	<0.0001
16	T6-hoof cranio-caudal distance protracted fore (mm)	0.18	0.03	(0.1, 0.2)	<0.0001	<0.0001
17	T6-hoof cranio-caudal distance retracted fore (mm)	0.14	0.03	(0.1, 0.2)	<0.0001	0.0002
18	S3-hoof cranio-caudal distance supporting hind (mm)	-0.04	0.02	(0.1, 0.0)	0.11	0.03
		Combined kinetic model				
19	Intercept	-4.32	2.78	(-9.8, 1.1)	0.17	<0.0001
20	Forelimb stance duration (ms)	-0.17	0.08	(0.1, 0.0)	0.04	0.01
21	Hindlimb stance protraction (mm)	-0.16	0.06	(0.1, 0.3)	0.02	0.001
22	Forelimb stance retraction (mm)	0.21	0.05	(0.1, -0.1)	0.0001	0.0002
23	Hindlimb stance retraction (mm)	0.21	0.07	(0.1, -0.1)	0.005	0.001
24	Ipsilateral limb tracking (mm)	-0.06	0.02	(0.1, 0.0)	0.005	0.01
		Combined kinetic/kinematic model				
25	Intercept	-2.22	1.69	(-5.5, 1.1)	0.24	<0.0001
26	Forelimb stance duration (ms)	-0.06	0.07	(0.1, 0.1)	0.40	0.03
27	Forelimb stance retraction (mm)	0.15	0.04	(0.1, -0.1)	0.001	0.0002
28	Hindlimb stance retraction (mm)	0.17	0.05	(0.1, -0.1)	0.002	0.0003
29	T6-hoof global distance protracted fore (mm)	0.30	0.11	(0.1, 0.5)	0.009	0.002
30	T6-hoof global distance retracted fore (mm)	-0.34	0.13	(-0.6, -0.1)	0.01	<0.0001
31	T6-hoof cranio-caudal distance protracted fore (mm)	0.15	0.04	(0.1, 0.2)	0.0009	0.0002
32	S3-hoof cranio-caudal distance supporting hind (mm)	-0.12	0.03	(0.1, -0.1)	0.002	0.0002

Est- estimate; 95% CI- 95% confidence interval

Untransf;-untransformed outcome

Transf;-transformed outcome

SD; stride duration

T6- sixth thoracic vertebra

S3- third sacral vertebra

In all analyses, there are 7 horses and each horse contributes data from between 5 and 8 trials (n = 37 trials for most analyses). The outcome is the difference between T6 vertical minimum in early left forelimb and right forelimb stance phases. Evaluated independent kinematic variables (last models in the table) are similarly left/right differences evaluated at the time of minimum vertical T6 position. P-values are shown for the presented-untransformed (Untransf.) models and transformed models (Transf., model estimates not shown).

The spatiotemporal model including stance lengths and durations (model i) showed that left-right differences in forelimb and hind limb stance lengths and forelimb stance durations had a significant influence on T6minDiff. Interpreting the coefficients, the positive estimate for forelimb stance length implies that a relative increase in LF stance length compared to RF stance length leads to a larger T6minDiff, which equates to a more pronounced dropping of the withers at the beginning of RF stance. The effect of forelimb stance length difference was about twice as large compared to the effect of hind limb stance length difference. Forelimb stance duration showed the opposite relationship to the findings for forelimb stance length: i.e. relatively longer stance duration in the RF was associated with a lower T6min in early stance of the RF. (Forelimb stance duration was non-significant in the model made using untransformed data, but the p-value from the model on the transformed scale was 0.02, see Table 1).

In the model describing fore- and hind limb protraction and retraction at the start and end of stance (model ii), differences in hind limb protraction, hind limb retraction, and forelimb retraction were significant. Withers drop in early RF stance increases when there is less protraction and retraction of the RH and more retraction of the LF.

The inter-limb coordination model (model iii) suggests that with increasing duration of the period of bipedal support by the ipsilateral LF and LH, T6minDiff increases (the withers drop more in early RF stance).

In the models with timing of force peaks (model iv) or magnitudes of the force peaks (model v) no variables were significant.

The model describing tracking of the hind limbs relative to the forelimbs (model vi) shows a negative estimate. This result suggests that, when one or both hind limbs track to the left of the respective forelimb (i.e. the RH is placed more under the body and the LH is placed lateral to the LF), this is associated with a larger withers drop in early RF stance (increases T6minDiff).

The kinematic model (Table 1, Fig 4) includes fore- and hind limb protraction-retraction position (distance from T6 to the hoof along the longitudinal axis) and global forelimb flexion-extension (distance T6-hoof) at the time of T6min. This model suggests that both forelimbs are relatively more flexed/compressed when T6min is lower. Note the opposite signs for estimates because the LF is retracted (further caudally) at RF T6min. Regarding the limb protraction/retraction positions, a lower T6min will result if the forelimb that is in early stance is more protracted and the forelimb in late stance is more retracted. Additionally, there was a slight decreasing effect on T6min if the ipsilateral hind limb was more retracted.

The typical limb patterns of protraction and retraction at T6min are illustrated in Fig 4. At the lower T6 minimum, the forelimb in early stance is more protracted and the forelimb in late stance is more retracted compared to the opposite step. This results in greater forelimb spread at the moment when T6 is lowest. According to the model, but not evident in the particular horse in Fig 4, the hind limb ipsilateral to the forelimb in early stance when T6 is lowest is slightly less protracted than the other hind limb in the opposite step.

## Discussion

This study investigates asymmetry in vertical minima of the withers during walking. Asymmetry of the vertical minima is associated with left-right differences in forelimb pro- and retraction and greater compression of both forelimbs at the time of occurrence of the minima (Fig 4). While one forelimb is progressively loaded in early stance the contralateral forelimb is retracted further and more quickly. This leads to a greater longitudinal distance between the two forelimbs, which makes the withers drop more. Concurrently, the haunches deviate away from the side of the forelimb that is in early stance when the withers are lowest. Thus, the ipsilateral hind limb tracks closer to the midline of the body. This hind limb also has a smaller range of protraction-retraction during stance. At ground contact the contralateral hind limb is placed lateral to the forelimb (Fig 3) and undergoes more protraction and more retraction during stance than the other hind limb, resulting in an overall increase in limb ROM in pro-retraction. Thus, the asymmetry in vertical movement of the withers is associated with a pattern of asymmetry in limb protraction and retraction and lateral displacement of the haunches relative to the forehand.

### Is this motor laterality?

In this study, 5/7 horses dropped the withers more in early LF stance, which was associated with a negative T6minDiff; one horse was reasonably symmetrical; and one horse dropped the withers more at early RF stance, which was associated with a positive T6minDiff (Fig 3). In classic horse training, it has been observed that most horses are more willing when lunged to the left than to the right, and many horses are reluctant, especially at first, to be lunged to the right at all [24]. This observation was supported by a study of motor laterality in young riding horses through an analysis of derailment during trot on circles [25]. In that study 70% of two-year-olds showed derailment when moving to the right, but none showed derailment to the left. Apart from behavioural observations, there are also biomechanical studies that have indicated that a majority of adult riding horses use the forelimbs differently. Horizontal moments around the center of pressure of the hooves of horses walking on a straight line were asymmetric with an internal moment around LF in all horses and an external moment around the RF in the majority (7/9) of horses [15]. When walking in circles, the internal moment around the LF was maintained in 5/6 horses when turning left and in 5/6 when turning right. In the RF, 4/6 exhibited an internal moment on the left circle and 5/6 had a weak external moment on the right circle [17]. The authors suggested that this was a consequence of the LF being actively retracted and internally rotated by the extrinsic musculature, while RF would be less actively retracted and would function more as a passive strut. This explanation agrees with the observations in the present study. In 5/7 horses, RF was retracted further and more rapidly than LF. Observations of video data (S1 Video) from the experiment give the impression that the horse vaults over the RF and then drops onto LF. If the withers drop further in early LF stance, there seems to be a quicker transition of weight-bearing between the two forelimbs when the horse shifts from RF to LF compared to shifting from LF to RF.

A risk factor for development of asymmetric hooves is asymmetrical grazing behaviour in foals, which is mainly prevalent in long-legged riding horses [14]. However, this does not explain the apparent population asymmetry bias unless the choice of limb positions is due to laterality of the cerebral hemispheres. This type of systematic cerebral laterality is present across a wide range of species including humans and, in some species, is associated with a left-right bias at population level [10].

### Are walk asymmetries reflected in trot?

All horses were deemed to be clinically sound at trot at the time of the study, though some individual horses showed vGRF asymmetries that were outside the 95% reference interval of 1.8–6.8% reported for vGRF parameters in sound horses at the trot [19]. In our group of horses, there was no consistent association between asymmetry in vGRF at the trot and vertical withers movement at the walk, though the variables are not necessarily wholly uncorrelated (Fig 2). In a study by Wiggers et al. [18] forelimb dorsal hoof angle asymmetry was correlated with asymmetrical forelimb loading at the trot. In that study 27/34 horses had uneven feet, defined as a left-right difference in dorsal hoof wall angles >1.5 degrees, with the flatter foot having significantly larger peak braking and peak vertical GRFs and a more supple fetlock spring, whereas the more upright foot had an earlier transition from braking to propulsion. In that study, 15 horses had a higher RF hoof and 12 horses had a higher LF hoof (Hobbs, personal communication). It was also reported that 8/27 of these horses were deemed sound at trot, but showed grade one lameness at walk, which was described as a ‘mechanical lameness’ because it was not thought to be pain-related. The movement asymmetry observed in walk, but not in trot [18], could represent the same phenomenon that is presented here, but direct comparisons cannot be made since hoof angles were not measured in our study.

### The equestrian perspective

When evaluating a horse’s gait, veterinarians are primarily focused on pain-related or pathological asymmetries. Riders and trainers are additionally concerned with asymmetries that are considered inherent to the horse, but can become relevant as they might limit performance. During a lameness examination, evaluation from the front and from behind are important components, whereas trainers and judges typically put more emphasis on the lateral view in which withers asymmetry is more readily appreciable. In the training situation laterality of horses is widely talked about, but to the best of the authors’ knowledge, the associated biomechanical asymmetries have not been quantified. In this context, the current study can be seen as one of the first steps to correlate objective measures with the rider’s perception and to bring more science into an area that has hitherto largely been dominated by empiricism.

### Benefits and limitations of the analysis strategy chosen

The study has some limitations. The number of horses was small. Walking on the treadmill may differ from over-ground walking. More specifically in relation to this project, the treadmill may help horses to maintain a straighter alignment thus contributing to within-horse consistency. Some of the findings or effects described in this study may be more difficult to observe when walking over ground due to a more variable motion pattern. Further, marker placement errors and skin displacement artefacts [26,27] are well-recognized, confounding factors in kinematic studies. However, the visual similarity of the motion patterns of T6, T10, T13, and the tubera spinae scapulae suggest that skin displacement or asymmetric marker misplacement were unlikely to explain the asymmetry pattern that was exhibited.

A parsimonious choice was made not to present speed-corrected models. When speed was forced into the final models, model estimates and significances changed only slightly, albeit speed was significant in some models. Previous studies have also shown that even if the movements, per se, change with increasing speed, asymmetry variables change minimally [28–30].

## Conclusions

This study shows the existence of consistent and repetitive vertical movement asymmetry of the forehand during walking in high-level dressage horses judged to be clinically sound. Asymmetrical vertical motion of the forehand was systematically associated with contralateral differences in forelimb and hind limb protraction and retraction and in hindquarter deviation from the sagittal plane, but not with differences in vertical ground reaction forces. The extent to which this pattern of asymmetry in walk is characteristic of the equine population at large is not known. It is discussed how this asymmetry might be associated with inherent or acquired laterality and asymmetries in trot, as well as in relation to equestrian training. Future studies need to investigate the effect of inherent asymmetry in ridden horses moving both on straight and curved lines, and in horses known to be clinically lame.

## Supporting information

S1 Fig The marker set up. The markers used in the current study were those on the sixth, tenth and thirteenth thoracic vertebrae (T6, T10, T13), third sacral vertebra (S3), spina scapula (3), elbow joint space (5) and lateral fore and hind hoof walls (not numbered).
(TIF)

S2 Fig Raw data for vertical positions of T6, T10, T13 and means of the vertical positions of the tubera spina scapulae markers for the trials in median speed for each horse. The curves showing vertical positions are centered around zero. For the ground reaction forces, the upper tracks are for the forelimbs and the lower tracks are for the hind limbs; in both cases the left limb is shown in blue and the right limb in red. The hind ground reaction forces are plotted with a negative offset of 5 N/kg.
(TIF)

S3 Fig Stance minimum vertical positions of T6 plotted against stance minimum vertical positions of other midline and forelimb markers evacuated from the time point during early stance of the left forelimb (only values for left forelimb stance are plotted to avoid stride-level clustering within trial). Minimal vertical positions for markers on the tuber spinae scapulae and elbow represent the mean of the left and right minima calculated on a stride-by-stride basis. Plots are shown for T6 versus T10 (top left), T13 (top right), and mean of the vertical position of the left and right tuber spinae scapulae (bottom left) and elbow joint (bottom right). Values inserted in the plots are median and range for Pearson trial-level correlations. Each horse is represented by a different color: Horse 1: blue; Hors 2: magenta; Horse 3: black; Horse 4: green; Horse 5: red; Horse 6: cyan; Horse 7: yellow.
(TIF)

S4 Fig Distributions of trial-level models independent data tested in kinetic and kinematic models. All variables are expressed as differences between left and right. Data—see S1 Table.
(PDF)

S1 Table The data used for the statistical analysis. (XLSX)

S1 Video Video of horse 2. Original speed 1.58 m/s; shown at 50% of original speed.
(MP4)
