# Supplementary figures and images for: Biomechanical findings in horses showing asymmetrical vertical excursions of the withers at walk
Source: PLoS One. 2018 Sep 27;13(9):e0204548. doi: 10.1371/journal.pone.0204548 (PMC6160136; doi:10.1371/journal.pone.0204548)

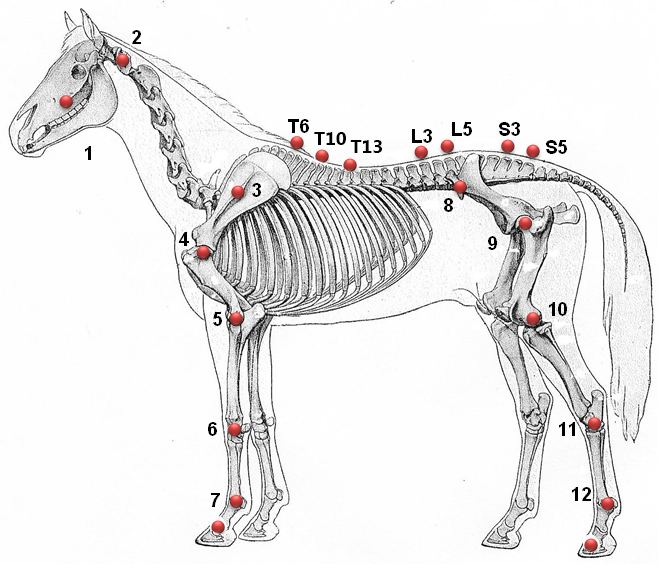

Supplement: S1 Fig — The markers used in the current study were those on the sixth, tenth and thirteenth thoracic vertebrae (T6, T10, T13), third sacral vertebra (S3), spina scapula (3), elbow joint space (5) and lateral fore and hind hoof walls (not numbered). (TIF) [file pone.0204548.s001.tif]

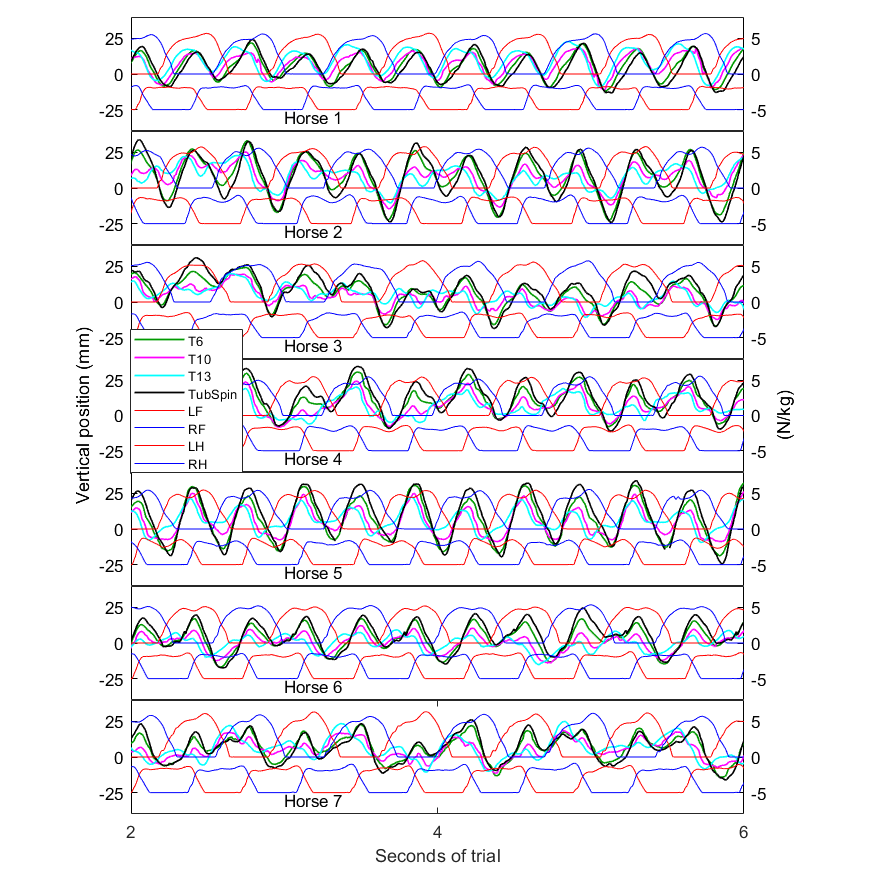

Supplement: S2 Fig — The curves showing vertical positions are centered around zero. For the ground reaction forces, the upper tracks are for the forelimbs and the lower tracks are for the hind limbs; in both cases the left limb is shown in blue and the right limb in red. The hind ground reaction forces are plotted with a negative offset of 5 N/kg. (TIF) [file pone.0204548.s002.tif]

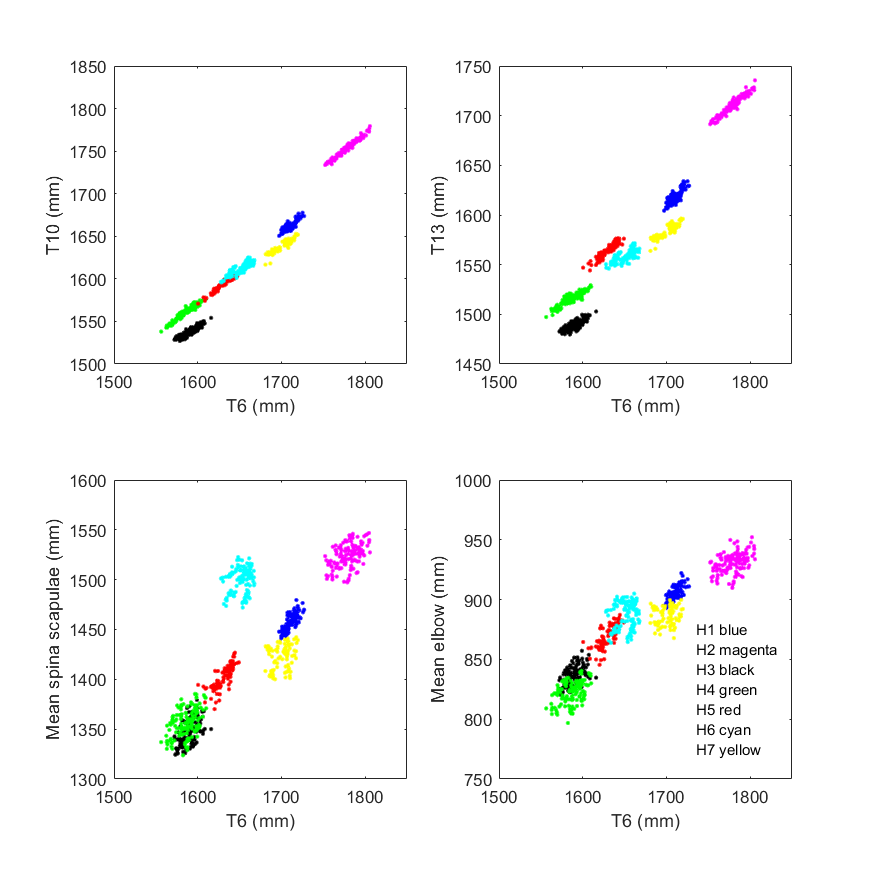

Supplement: S3 Fig — Minimal vertical positions for markers on the tuber spinae scapulae and elbow represent the mean of the left and right minima calculated on a stride-by-stride basis. Plots are shown for T6 versus T10 (top left), T13 (top right), and mean of the vertical position of the left and right tuber spinae scapulae (bottom left) and elbow joint (bottom right). Values inserted in the plots are median and range for Pearson trial-level correlations. Each horse is represented by a different color: Horse 1: blue; Hors 2: magenta; Horse 3: black; Horse 4: green; Horse 5: red; Horse 6: cyan; Horse 7: yellow. (TIF) [file pone.0204548.s003.tif]

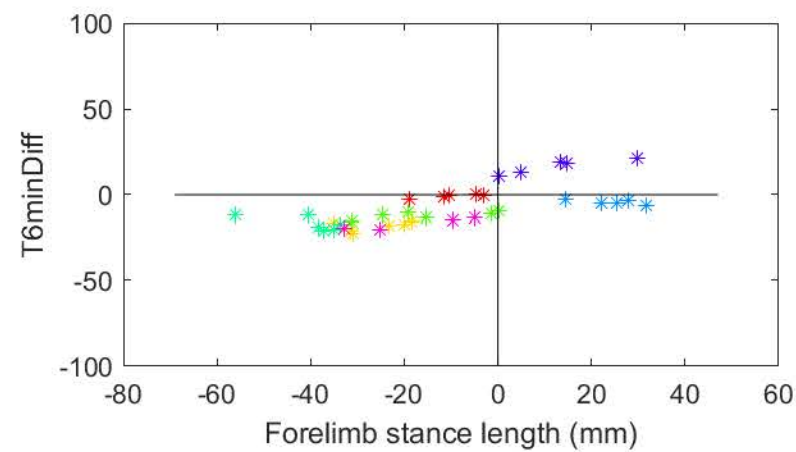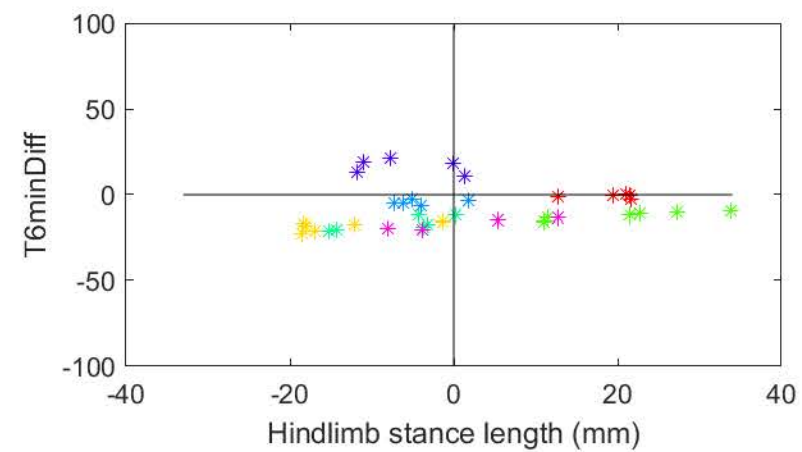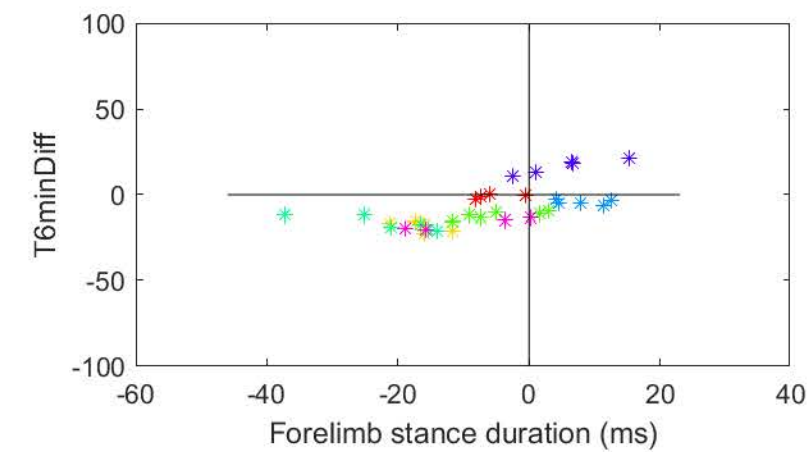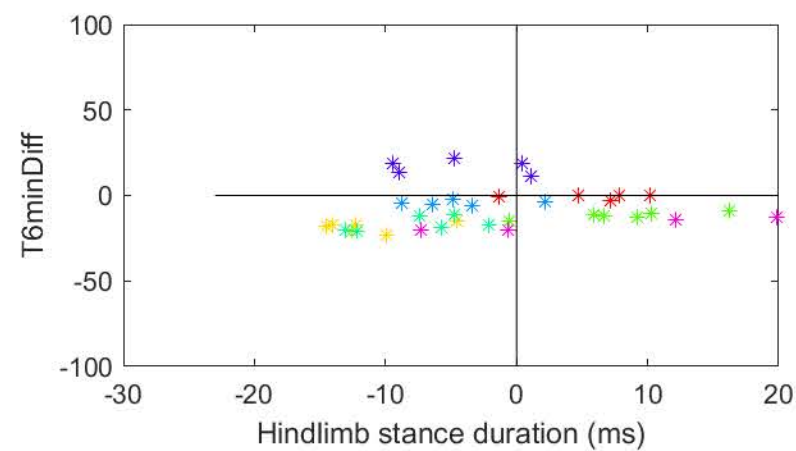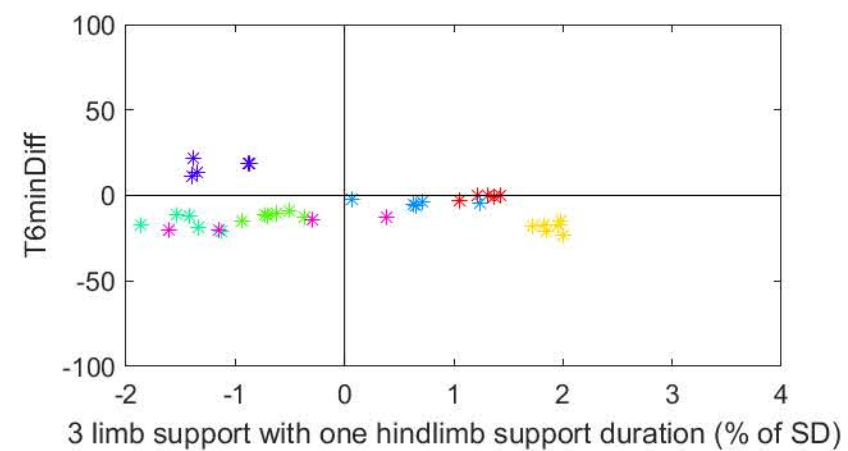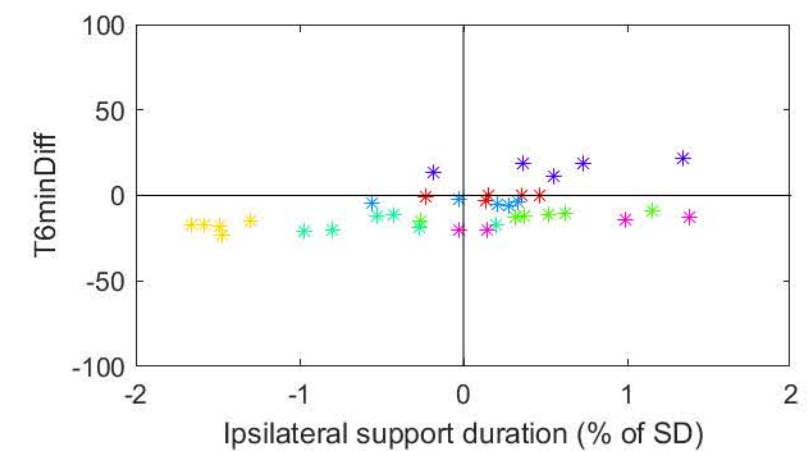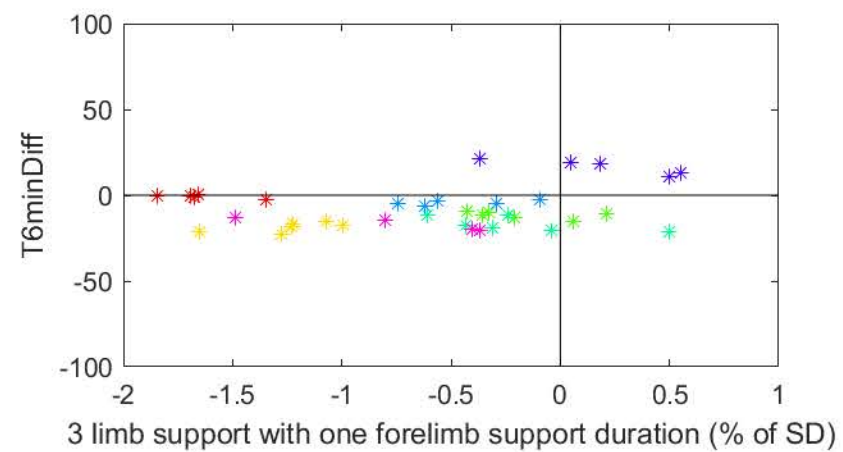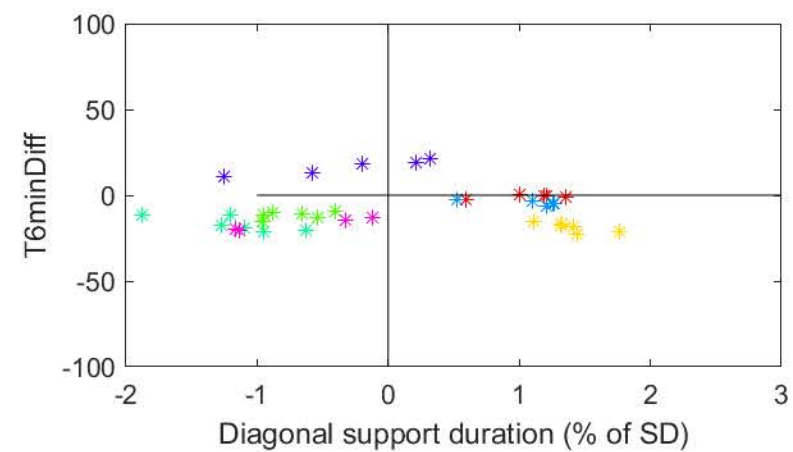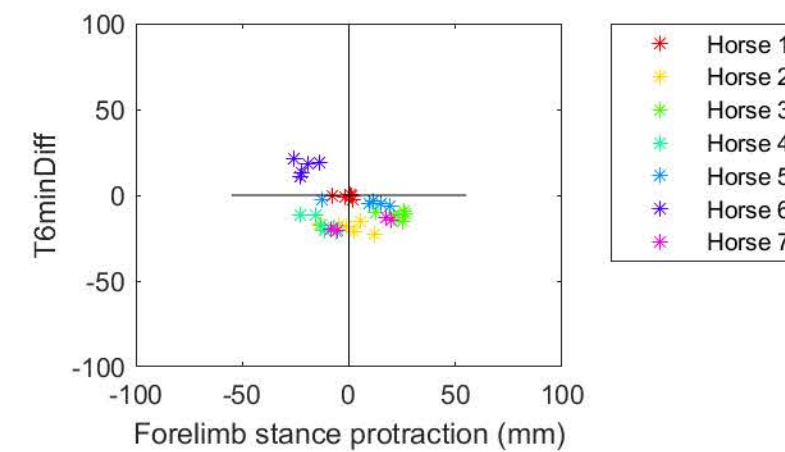

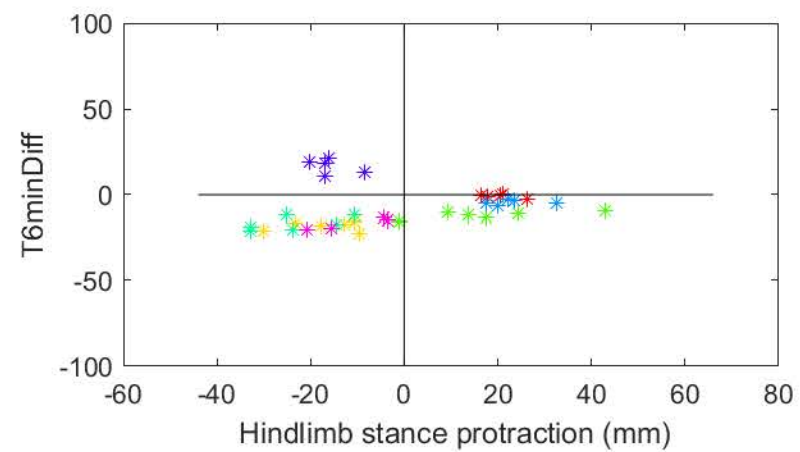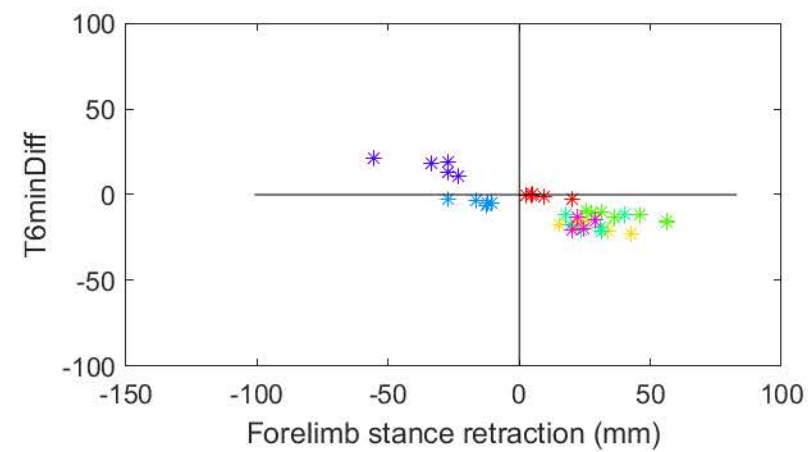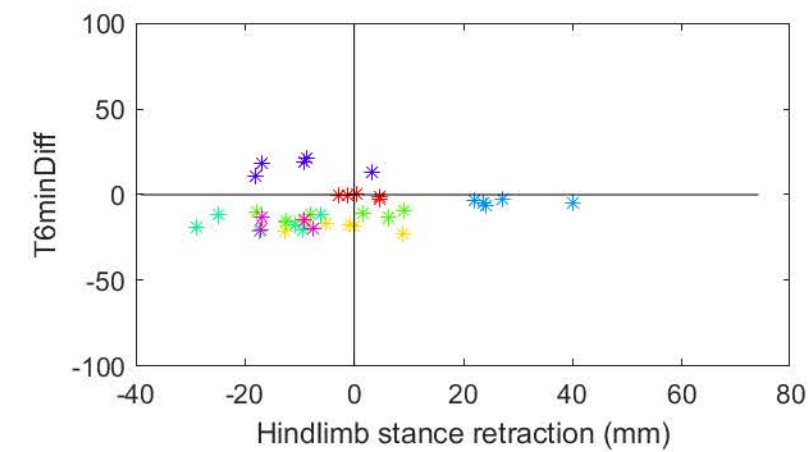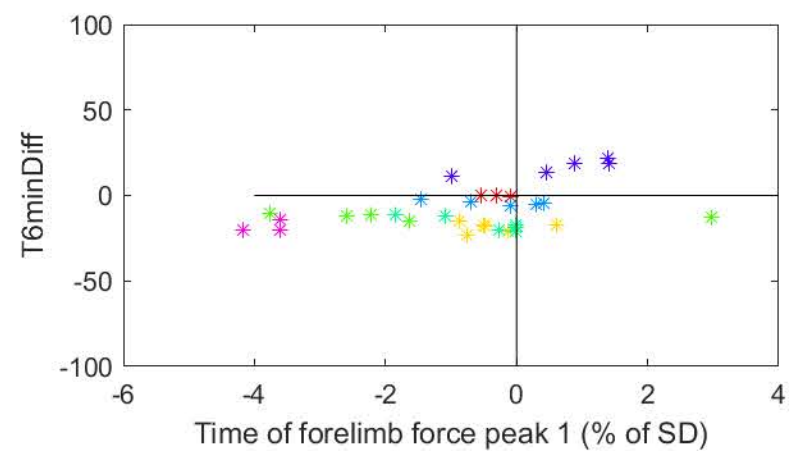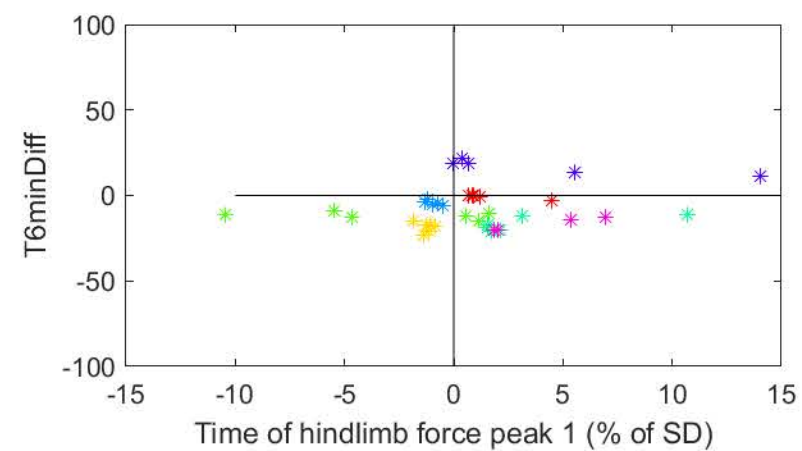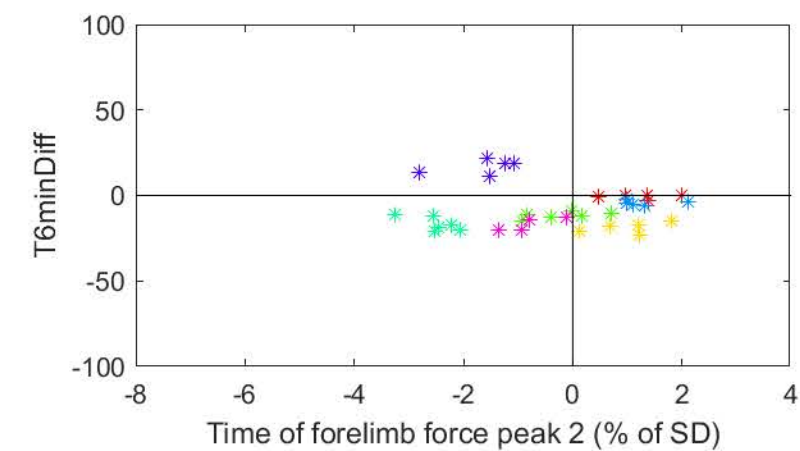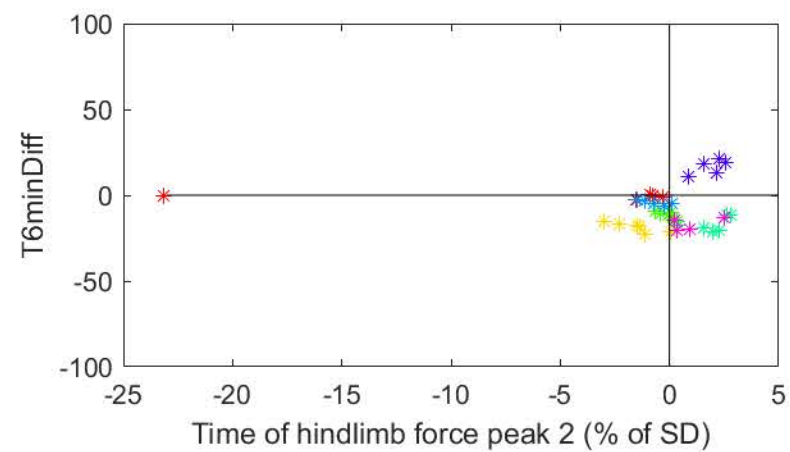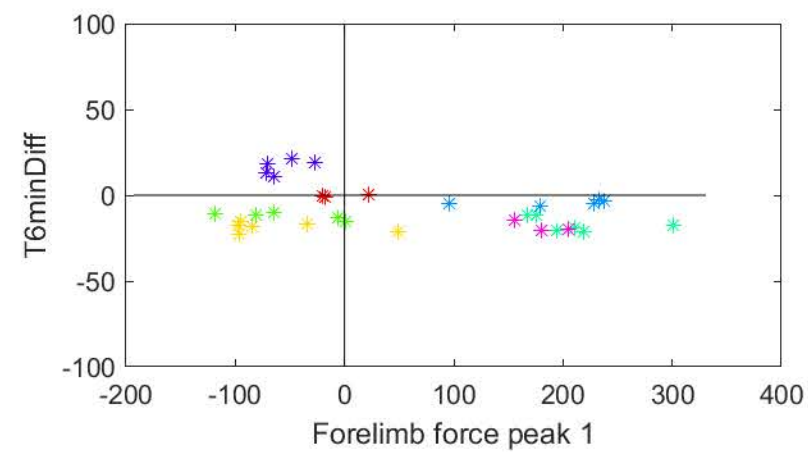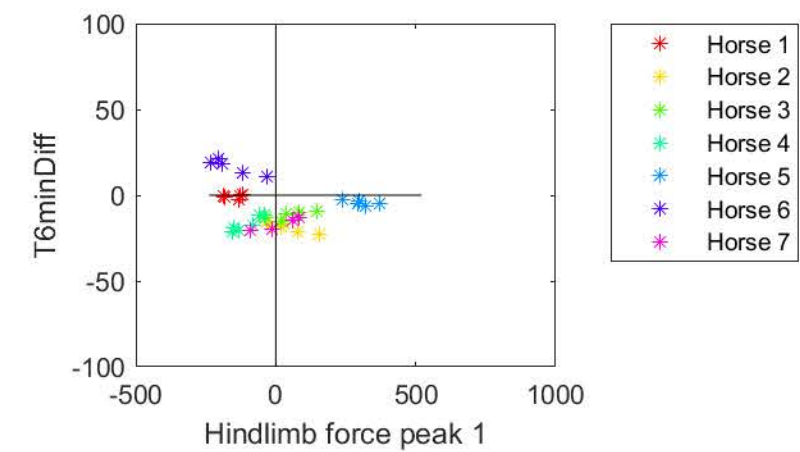

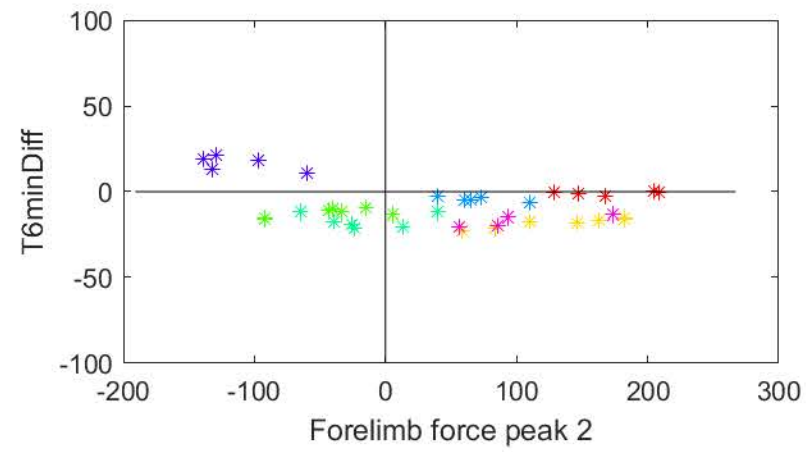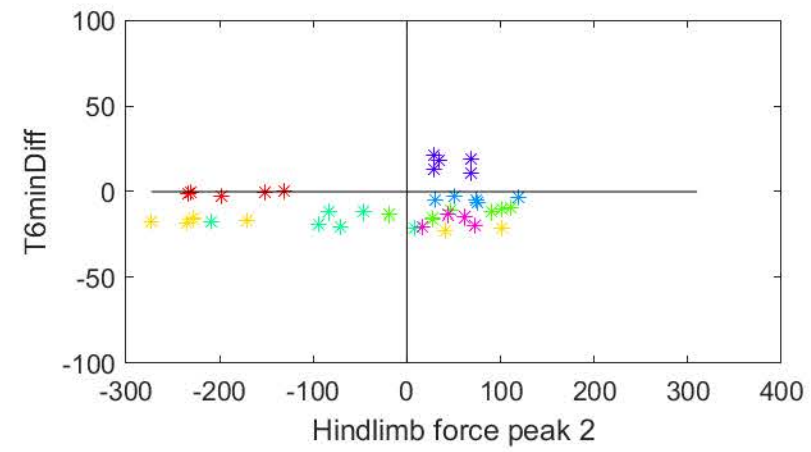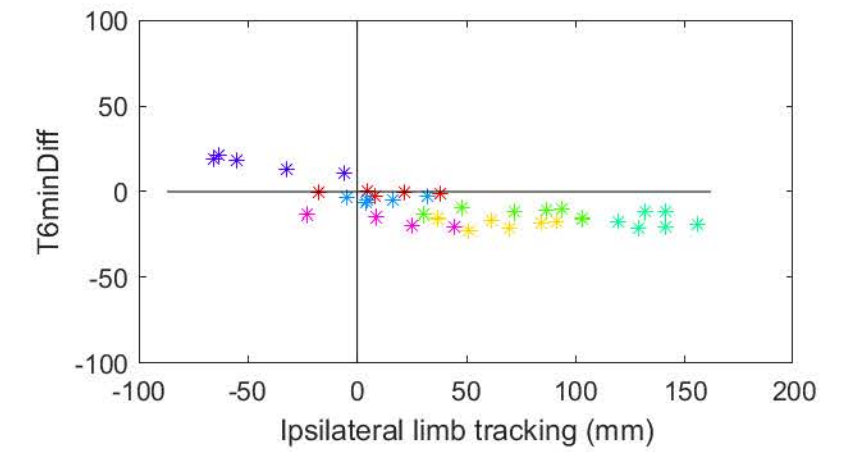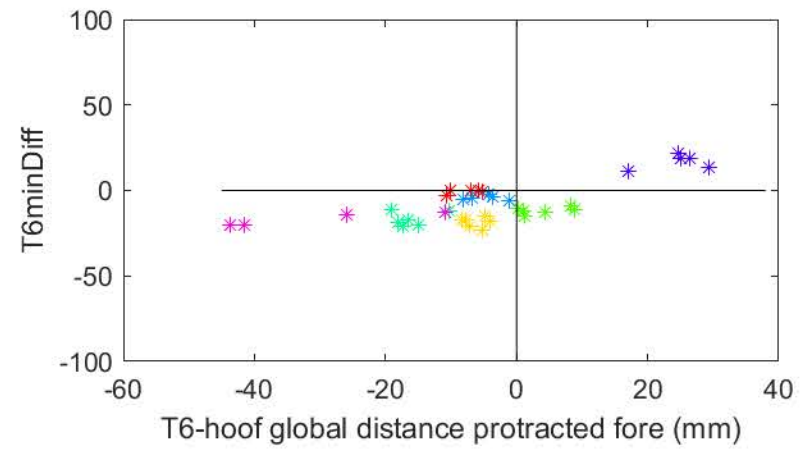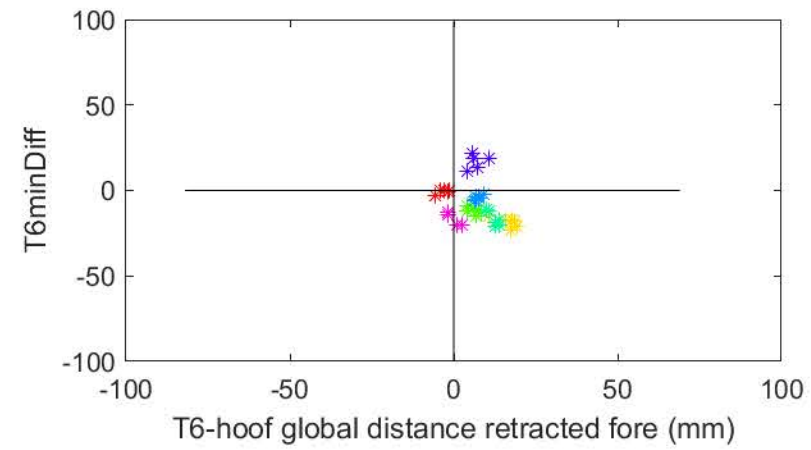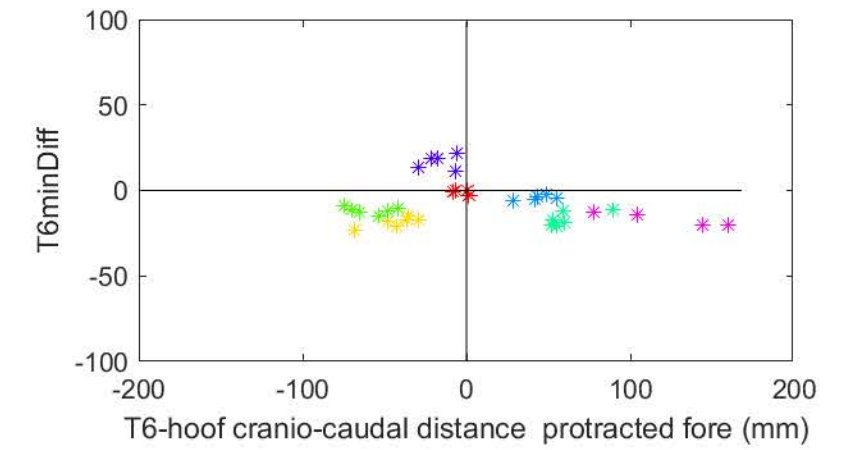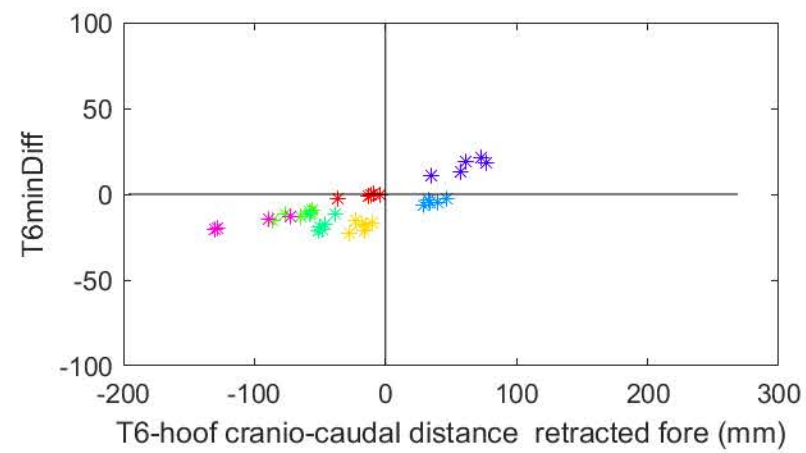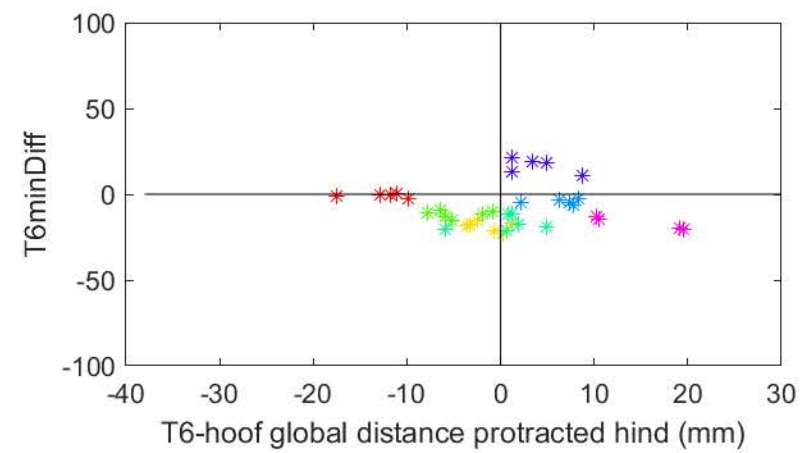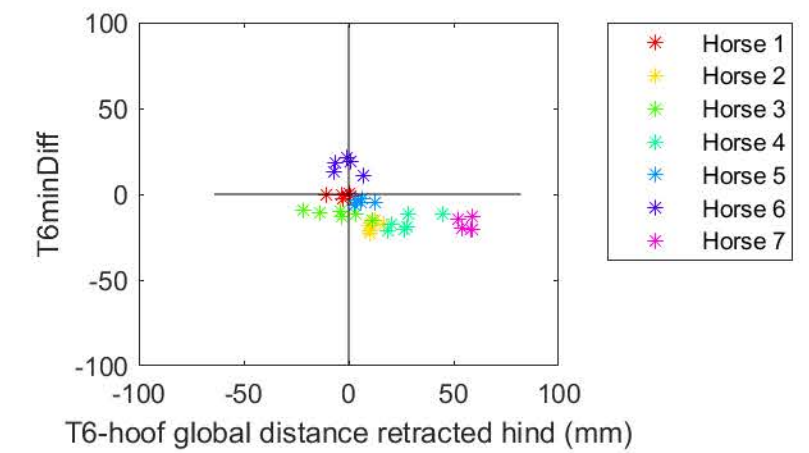

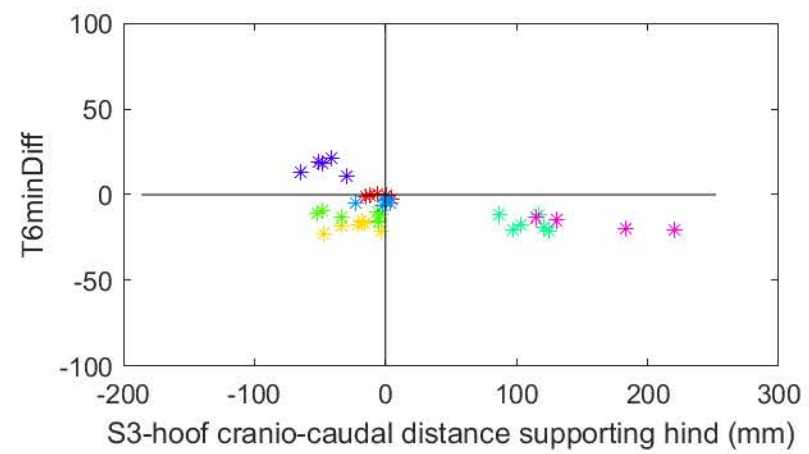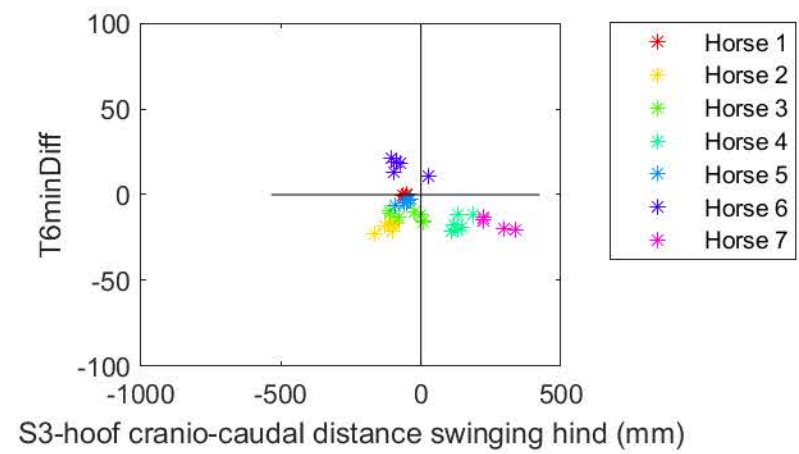

Supplement: S4 Fig — All variables are expressed as differences between left and right. Data—see S1 Table. (PDF) [file pone.0204548.s004.pdf]
